# Modeling the risk of airborne transmission of respiratory viruses in microgravity

**DOI:** 10.1038/s41526-026-00590-4

**Published:** 2026-04-01

**Authors:** Chayanin Sararat, Natnicha Jiravejchakul, Kawin Nawattanapaiboon, Charin Modchang

**Affiliations:** 1 Biophysics Group, Department of Physics, Faculty of Science, Mahidol University, Bangkok, Thailand; 2 Department of Microbiology, Faculty of Science, Mahidol University, Bangkok, Thailand; 3 Zenostic Co. Ltd, Bangkok, Thailand; 4 Centre of Excellence in Mathematics, MHESI, Bangkok, Thailand; 5 Thailand Center of Excellence in Physics, Ministry of Higher Education, Science, Research and Innovation, Bangkok, Thailand

**Keywords:** Biophysics, Viral infection, Applied mathematics

## Abstract

Airborne transmission is one of the most efficient routes of respiratory viral spread, posing a significant challenge in controlling major infectious diseases such as COVID-19. In microgravity environments, such as the International Space Station (ISS), this mode of transmission requires heightened vigilance and preventive measures due to the prolonged suspension of virus-laden particles, which increases the risk of infection. Using the COVID Airborne Risk Assessment (CARA) tool, we assess the risk of airborne transmission of respiratory viruses, using SARS-CoV-2 as a case study, in microgravity by simulating the emission, dispersion, and inhalation of virus-laden particles. Our simulations show that the unique conditions of microgravity allow these particles to remain airborne for more extended periods compared to Earth, leading to a 286-fold increase in virus concentration in the air and resulting in nearly twice the probability of infection for a susceptible host. We also evaluated the effectiveness of preventive measures. We found that facemasks could reduce the risk by up to 23%, while continuous HEPA filtration at five air changes per hour proves crucial for managing air quality and minimizing infection risks by reducing airborne virus concentration by 99.79%. To explore potential effects of spaceflight-induced immune suppression on transmission risk, we modeled hypothetical scenarios with increased viral shedding based on herpesvirus reactivation data. An 8-fold increase in viral load (as observed for herpesviruses in space) raised infection probability by 12 percentage points above baseline. Sensitivity analysis with 4-fold and 16-fold increases showed infection risk scales proportionally with viral shedding intensity. Although facemasks and air filtration help mitigate the risk, their effectiveness diminishes when viral load is elevated. Enhancing host immunity through vaccination or other interventions is vital, potentially reducing infection probability by up to 14.17% when combined with HEPA filtration.

## Introduction

The study of airborne transmission of respiratory viruses has gained significant attention in recent years, particularly in light of global health challenges such as the COVID-19 pandemic^1–3^. Understanding the dynamics of how these viruses spread in various environments is crucial for developing effective prevention and control measures. One area that remains relatively underexplored is the behavior of respiratory virus transmission under microgravity conditions, such as those experienced by astronauts aboard the International Space Station (ISS). Although the possibility of an astronaut carrying a pathogen and eventually causing an infection is small, once an infection does occur in microgravity conditions, the limited healthcare facilities on the ISS could exacerbate the situation, making it more challenging to manage and treat. Our hypothesis posits that in such conditions, respiratory particles emitted from a pathogen carrier could remain airborne for longer durations, thereby increasing the risk of transmission among astronauts.

Spaceflight creates a uniquely stressful environment for astronauts^4^. In addition to the effects of microgravity, astronauts encounter various stressors, including confinement in an unfamiliar environment, isolation, separation from family, sleep deprivation, noise, and anxiety. These factors have the potential to weaken the immune system during missions^5^. Research has shown significant alterations in immune cell numbers^6,7^, their functions^6–8^, development^6–8^, and cytokine secretion^6,9^. These changes suggest that the immune system may be compromised during spaceflight, potentially increasing the host susceptibility and reactivation of asymptomatic and latent viral infection.

One notable indicator of immune system degradation in space is the reactivation of herpesviruses^10,11^. It was estimated that up to ninety percent of the human population, including most astronauts, is infected with herpesviruses^12^. These viruses typically remain in a latent or dormant state after the initial infection and are usually asymptomatic in individuals with a competent immune system^13^. However, herpesvirus reactivation has been documented even in astronauts who are in excellent health and superb physical condition^5,10,12,14–16^. This suggests a potential immune system compromise, which might increase the overall risk of disease among crew members.

In the context of respiratory disease transmission, the unique microgravity environment aboard the ISS may further exacerbate the risk. Under normal gravity conditions on Earth, respiratory particles expelled by an infected individual tend to settle relatively quickly due to gravitational forces^17,18^. However, in the microgravity environment, these particles may remain suspended in the air for extended periods, increasing the possibility of inhalation by other crew members. This prolonged suspension, coupled with the confined living quarters and the recirculation of air, could create conditions conducive to the spread of respiratory viruses. Investigating the potential risks associated with this unique environment is essential for developing effective strategies to mitigate the transmission of infectious diseases during spaceflight, where medical facilities are limited.

To address these concerns, a comprehensive risk assessment of airborne transmission under microgravity conditions is essential. While several studies have examined immune changes in spaceflight, the specific implications for respiratory virus transmission dynamics in microgravity remain largely unexplored. This represents a critical knowledge gap in space medicine and biosafety. Understanding how the unique physics of particle behavior in microgravity alters transmission risk and evaluating the effectiveness of terrestrial control measures in this environment would enable the development of targeted strategies to mitigate the spread of respiratory viruses aboard the ISS. Beyond enhancing astronaut safety, such knowledge has broader applications for understanding infectious disease transmission dynamics in other confined spaces and extreme environments where air circulation patterns and particle behavior may be altered.

In this study, we conduct a theoretical investigation to quantify the risk of airborne transmission of respiratory viruses under microgravity conditions, using SARS-CoV-2 as a case study. We adapt the validated COVID Airborne Risk Assessment (CARA) tool^1^ by modifying key parameters to simulate microgravity environments, particularly the gravitational settling velocity of respiratory particles and the subsequent changes in particle suspension time. Our computational model simulates the entire transmission pathway: from emission of virus-laden particles, through their dispersion in the ISS environment, to inhalation by susceptible hosts. We further examine how spaceflight-induced alterations in host immunity might influence viral shedding and transmission risk and evaluate various mitigation strategies, including facemasks and High-Efficiency Particulate Air (HEPA) filtration. The insights from these simulations could help inform evidence-based infectious disease management protocols for future space missions, addressing the unique challenges posed by the spaceflight environment and enhancing crew safety during long-duration missions.

## Results

### Risk of airborne transmission under microgravity

**Fig. 1:**
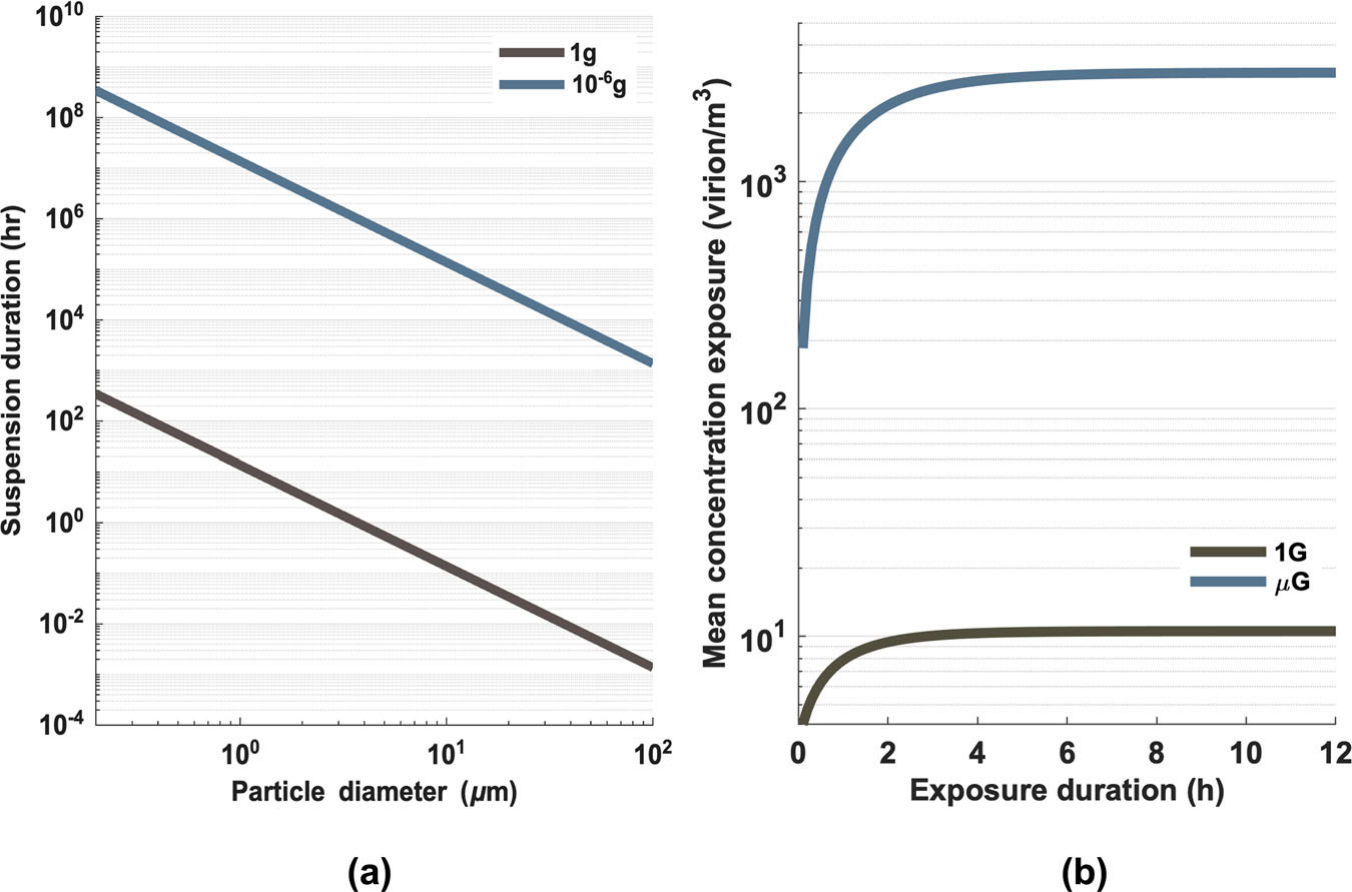
Impact of microgravity on respiratory droplet behavior and viral concentration. **a** Comparison of respiratory droplet suspension duration in Earth’s gravity (*g* = 9.8 m/s²) versus microgravity (*g* = 9.8 × 10^−6^ m/s²), illustrating the dramatically increased airborne time in reduced gravitational conditions. **b** Temporal evolution of SARS-CoV-2 Delta variant concentration in an enclosed space containing an infected transmitter, contrasting Earth’s gravity and microgravity environments.

Low-gravity environments can cause particle behavior to differ from that observed on Earth. The reduced gravitational pull allows particles to remain suspended in the air for much longer periods than they would under Earth’s gravity. For example, a particle with a 3-µm diameter could theoretically stay airborne for 17.5 years (~1.5 million hours) under a gravity of 10^−6^ g, compared to just 1.5 h under Earth’s gravity (Fig. 1a). Particles as large as 10 micrometers, which typically settle within 8 min on Earth, could remain suspended for up to 15.7 years in microgravity. This prolonged suspension time could contribute to the accumulation of virus-laden particles in confined spacecraft environments, creating a fundamentally different airborne transmission dynamic than observed in terrestrial settings and potentially leading to an increased risk of transmission between individuals or across spacecraft compartments.

**Fig. 2:**
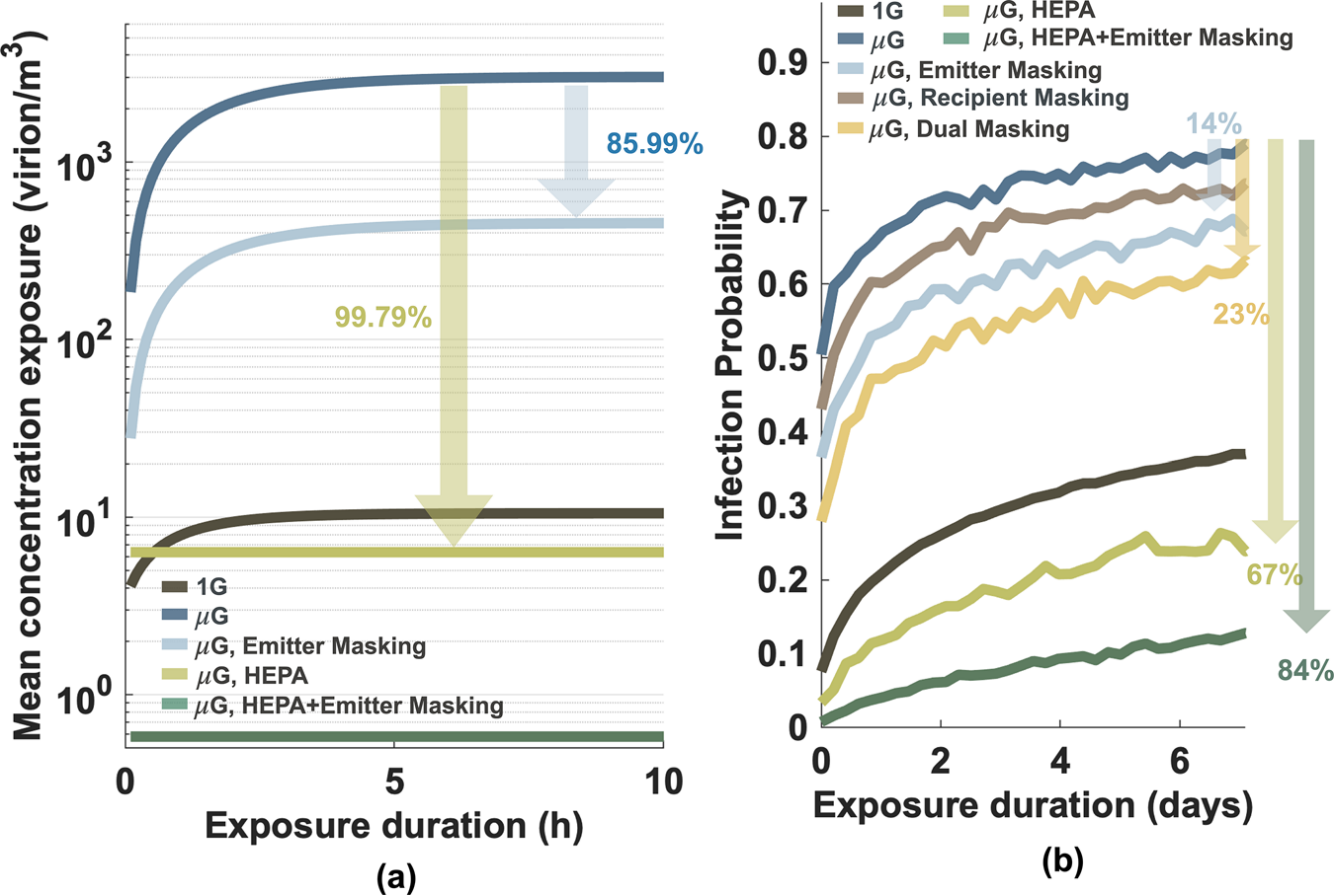
Efficacy of masking and HEPA filtration in mitigating viral transmission under microgravity conditions. **a** Reduction in airborne viral concentration and **b** decrease in infection probability over a 7-day exposure period under various intervention scenarios. Simulations evaluate mask-wearing by the emitter, recipient, or both, as well as HEPA filtration alone and in combination with emitter masking.

Our simulation results, in the absence of precautionary measures, suggested that the concentration of viruses in the ambient air in microgravity environments could increase by approximately 286-fold, from 10 virions/m³ under Earth’s gravity to 3013 virions/m³ in microgravity (Fig. 1b). As a result, after one week of exposure, the probability of infection of the virus in a microgravity environment could reach 78%, nearly double the infection probability under Earth’s gravity (Fig. 2). Given these elevated risks, we evaluated several potential mitigation strategies. We found that wearing a facemask reduced the number of virus-laden droplets released into the air by 84.99% (Fig. 2a). In this source control scenario, the infection probability decreased to 67% (Fig. 2b), representing a 14% reduction. Notably, this approach was found to be more effective than when only the susceptible host wore a mask, where the infection probability remained at 72%. The infection probability further decreased to 60% when both the susceptible and infectious hosts wore masks.

Viruses adhered to particles can be effectively removed from the air when trapped within filter media, thereby decreasing the overall viral load in the environment. In the context of spacecraft environmental systems, High-Efficiency Particulate Air (HEPA) filters, operating continuously at 5 air changes per hour (ACH), which is comparable to standards in biological safety laboratories and hospital wards^19^, could reduce the steady-state concentration of airborne viruses by 99.79% (Fig. 2a). This reduction achieves concentrations even lower than those observed under Earth’s gravity. With HEPA filters in place, the infection probability could be reduced to 25%, which is lower than the infection probability under Earth’s gravity, highlighting the importance of effective filtration systems in mitigating the risk of airborne transmission in microgravity environments.

### Effects of suppressed host immunity during spaceflight

The stress and altered conditions experienced during spaceflight can cause significant changes in immune response^6–8^, including reduced activity of T-cells^6,8^, which play a crucial role in fighting viral infections. Spaceflight has been shown to alter the production of cytokines^6,20^, the signaling molecules that mediate immune responses, potentially leading to impaired immune regulation and response. Previous studies have shown that reactivation of herpesviruses in astronauts occurs significantly more frequently during spaceflight, with viral shedding increasing by approximately 8-fold compared to Earth conditions^14^.

While we recognize that the replication dynamics and shedding behavior of coronaviruses differ substantially from those of herpesviruses, these findings nevertheless provide empirical evidence that spaceflight-induced immune suppression can markedly enhance viral reactivation and shedding. Given the absence of direct data on SARS-CoV-2 behavior in space, we adopted a modeling approach that explores a range of hypothetical scenarios. We used the 8-fold increase observed in herpesviruses as a reference point, not to suggest biological equivalence, but to establish a plausible estimate for potential risk assessment. To address the uncertainty inherent in this extrapolation, we also conducted sensitivity analyses with viral load increases of 4-fold, 8-fold, and 16-fold relative to baseline conditions.

**Fig. 3:**
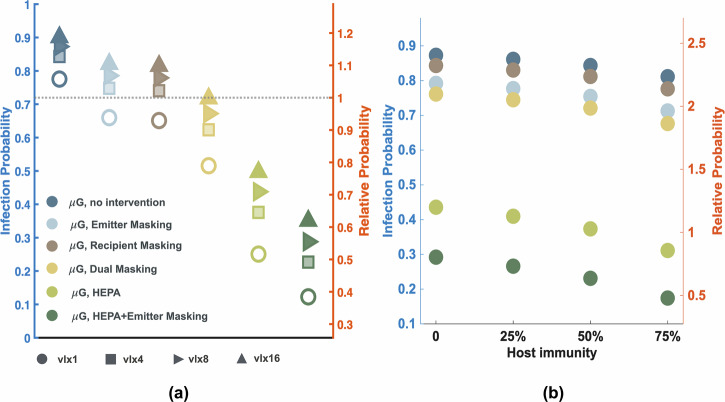
Impact of viral load and host immunity on infection probabilities in microgravity over a 7-day exposure period. **a** Infection probabilities under different viral load scenarios (1×, 4×, 8×, and 16× baseline) and intervention strategies in microgravity (μG). Interventions include emitter masking, recipient masking, dual masking, HEPA filtration alone, and HEPA filtration combined with emitter masking. The horizontal dotted line indicates the baseline infection probability under Earth gravity without interventions. **b** Effect of host immunity levels (0%, 25%, 50%, and 75%) on infection probability under the 8-fold viral load scenario. For both panels, the left y-axis shows absolute infection probability, and the right y-axis shows relative probability compared to the baseline transmission under Earth gravity without any measures.

Under the 8-fold viral load scenario, our simulations showed that infection probability reached 87%, representing an increase of approximately 12% compared to baseline conditions in the absence of protective measures (Fig. 3a). Even with masks worn by both the transmitter and susceptible individual, infection risk remained substantially elevated. Furthermore, HEPA filtration alone resulted in a 43% transmission risk under this elevated viral load—still 19% higher than observed under Earth’s gravity without interventions.

Our sensitivity analysis revealed a dose-response relationship between viral load and infection probability. With a 4-fold increase in viral load, infection probabilities reached 85% without protective measures (9% above baseline), and HEPA filtration successfully reduced the risk to levels comparable to Earth gravity without interventions. At the other extreme, a 16-fold increase yielded a 90% infection probability without measures (16% above baseline). These results demonstrate that while our specific quantitative predictions depend on the assumed viral load multiplier, microgravity might indirectly influence SARS-CoV-2 transmission through immune system suppression, leading to enhanced viral shedding that complicates the control of airborne viral transmission during spaceflight.

Boosting host immunity through methods such as vaccination is crucial for reducing the risk of infection. Our findings demonstrated that a 50% increase in host immunity could reduce the infection probability by 3.4% without any other intervention, by 4.7% when the transmitter wore a mask, and by a substantial 14.17% when HEPA filtration was employed (Fig. 3b). The reduction achieved through HEPA filtration brought the infection probability down to a level comparable to our baseline scenario of transmission on Earth without any intervention. Furthermore, combining the use of a mask on the transmitter with HEPA filtration could further lower the infection probability, reducing it to a level even lower than the baseline scenario. These results highlight the importance of a multi-faceted approach to mitigating the risk of infection in the unique environment of spaceflight, where the combined effects of suppressed host immunity and prolonged particle suspension can significantly increase the likelihood of disease transmission.

## Discussion

Contamination during space flights, despite rigorous pre-launch screening, remains a possibility. Astronauts can harbor viruses and microbes in dormant or asymptomatic states, which may become active and shed later in space when the immune system is compromised. Additionally, even though astronauts are in space, the spacecraft itself is constructed on Earth and can carry viruses or microbes into space, independent of the crew^21,22^. Furthermore, if an infection does occur during space flights, the limited healthcare facilities onboard could make managing and treating the condition more challenging than on Earth.

In microgravity environments, the physics governing particle behavior fundamentally changes. Aerosols and droplets do not settle to the ground as they do on Earth, but can remain suspended in the air for extended periods. This prolonged suspension allows particles, including virus-laden droplets, to accumulate in the environment, potentially leading to increased viral concentrations. Our theoretical modeling suggests that this reduced gravitational force could lead to an approximately 286-fold increase in airborne viral concentration. Consequently, the simulation indicates that infection probability in a microgravity environment could reach 78% after one week of exposure, nearly double the infection probability under Earth’s gravity. These findings, while based on computational modeling rather than experimental evidence, highlight the potentially heightened transmission risks in spacecraft environments.

Our simulations suggest that preventive measures could substantially mitigate these risks. Facemasks appear to play a crucial role in reducing infection transmission in microgravity environments, with source control (having the infectious host wear a mask) being more effective than protection of the susceptible host alone. However, a significant limitation of our work is the assumption that mask filtration performance remains consistent in microgravity. In reality, the absence of gravitational settling and reduced natural convection currents might alter airflow patterns around masks and potentially affect their filtration efficiency^23^. This represents an important area for future experimental investigation to validate our theoretical predictions.

Similarly, HEPA filtration systems could be critical for managing air quality in spacecraft. Our simulations with HEPA operating continuously at five air changes per hour (ACH), comparable to standards in biological safety laboratories and hospital wards^19^, predict a 99.79% reduction in steady-state airborne viral concentrations. At the International Space Station (ISS), HEPA filtration media are integrated within the bacteria filter elements (BFEs). The airflow through these BFEs varies between modules to maintain optimal cabin ventilation characteristics^24^. For instance, in the U.S. Laboratory (Destiny), 6 BFEs handle an airflow of approximately 113 m^3^/h, while in Node 1 (Unity), 4 BFEs manage about 127 m^3^/h^24^. Given the respective volumes of 123 m^3^ for Destiny and 80 m^3^ for Unity^25^, this results in air change rates of approximately 6.5 ACH and 6.4 ACH, respectively. These rates exceed those used in our simulations, indicating a more rigorous air filtration process on the ISS, which further ensures the reduction of airborne viral concentrations and enhances overall air quality.

The potential interaction between microgravity and host immunity represents another critical dimension of infection risk. Studies demonstrate that spaceflight conditions alter immune function^26,27^, potentially leading to an immunocompromised state similar to that observed in certain patient populations on Earth^21,27–31^. Reactivation of latent viruses occurs more frequently during spaceflight^12–14^, with viral shedding approximately eight times higher than on Earth^14^.

In the absence of empirical data on SARS-CoV-2 behavior in space, we adopted a modeling approach that explores plausible scenarios rather than making definitive predictions. We used the well-documented 8-fold increase in herpesvirus shedding as an illustrative reference point, while fully acknowledging the fundamental biological differences between these viral families. Herpesviruses and coronaviruses differ markedly in their replication strategies, latency mechanisms, routes of infection, and host-pathogen interactions. Herpesviruses establish latency and reactivate from dormancy, while SARS-CoV-2 causes acute infections without true latency. Despite these differences, the herpesvirus data provide valuable empirical evidence that spaceflight conditions can substantially amplify viral shedding through immune suppression—a mechanism that could theoretically affect various pathogens, albeit to different degrees.

To account for this uncertainty, we modeled multiple scenarios with viral load increases ranging from 4-fold to 16-fold, treating the 8-fold value as a mid-range estimate for risk assessment purposes, rather than a biological prediction. Our simulations indicated that an 8-fold increase in viral load could raise infection probability by approximately 12%, potentially diminishing the effectiveness of preventive measures. Even with HEPA filtration systems in place, infection risk was projected to remain higher than under terrestrial conditions. The sensitivity analysis confirmed that while the exact quantitative risk depends on the magnitude of viral load increase, the qualitative finding that spaceflight-associated immune suppression could substantially compromise infection control measures remains consistent across the range of scenarios tested.

The immunological impacts of spaceflight could extend beyond latent virus reactivation. Studies suggest that microgravity is linked to accelerated immunosenescence and compromised barrier function^21,27,32^. Although SARS-CoV-2 is not a latent virus, its ability to cause asymptomatic infections with viral shedding^33^, along with the altered immune environment in space, could create unique transmission dynamics not observed on Earth. Additionally, other airborne pathogens with long incubation periods, such as *Mycobacterium tuberculosis*^34^, might pose similar or greater risks during extended missions.

Our modeling indicates that enhancing host immunity could be particularly valuable in this context. When combined with environmental controls like HEPA filtration, a theoretical 50% enhancement in host immunity could reduce infection probability to levels comparable to those on Earth without interventions. Multi-layered preventive strategies appear to have synergistic effects in our simulations. Given that vaccine effectiveness can be lower in immunocompromised populations (64% compared to 79% in immunocompetent individuals)^35^, specialized approaches to immune enhancement for spaceflight might be worth investigating.

This study has several important limitations that should be considered when interpreting our results. First, we adapted an established terrestrial model (CARA)^1^ to the space environment by modifying gravitational parameters, but the model itself has not been validated in actual microgravity conditions. Second, our assumption about increased viral load was based on herpesvirus data, as no SARS-CoV-2 data from spaceflight environments exists. The biological characteristics, infection mechanisms, and host-pathogen interactions differ significantly between these viruses, potentially limiting the applicability of this assumption. Third, we did not account for radiation effects, although the ISS does provide substantial radiation shielding^36^. Finally, we assumed consistent mask performance between Earth and microgravity environments, which requires experimental validation.

Another important consideration not fully addressed in our model is the potential for viruses to adapt to the space environment. Research on viromes in spacecraft is limited compared to bacterial and fungal microbiome studies^21,37^. The few available studies show complex effects: microgravity can inhibit some viruses like Kaposi’s sarcoma-associated herpesvirus^38^ while potentially facilitating viral spread through increased gut barrier permeability^32^. How SARS-CoV-2 or other respiratory viruses might behave or evolve in prolonged microgravity remains an open question.

In addition, two physical processes were simplified in the current framework. First, the efficiency of ventilation systems can vary substantially depending on the ventilation type. On Earth, displacement and mixing ventilation produce different airflow patterns and clearance rates, and in microgravity, where buoyancy-driven convection is absent, and forced ventilation predominates, these dynamics could behave differently. Second, the well-mixed assumption applied here does not capture elevated exposure in the near-field region immediately surrounding an infectious host. Short-range jets generated by breathing, speaking, or coughing may lead to higher localized concentrations than are reflected in room-averaged models. Future research integrating ventilation strategies and host-to-host proximity effects would therefore provide more accurate risk assessments.

In conclusion, our theoretical modeling suggests that microgravity environments could substantially increase the risk of respiratory virus transmission through extended particle suspension times and potential immune system alterations. While current environmental control systems on the ISS appear robust, additional preventive measures may be necessary, particularly for longer missions or smaller spacecraft with less sophisticated air management systems. Future research directions should include experimental validation of mask performance in microgravity, direct measurement of viral shedding patterns during spaceflight, and development of spaceflight-specific immune enhancement strategies. Understanding these complex interactions between microgravity, virus behavior, and host immunity will be crucial for safeguarding astronaut health during future exploration missions, particularly those beyond low Earth orbit, where rapid return to Earth is not possible.

## Methods

### Infection risk assessment

**Fig. 4:**
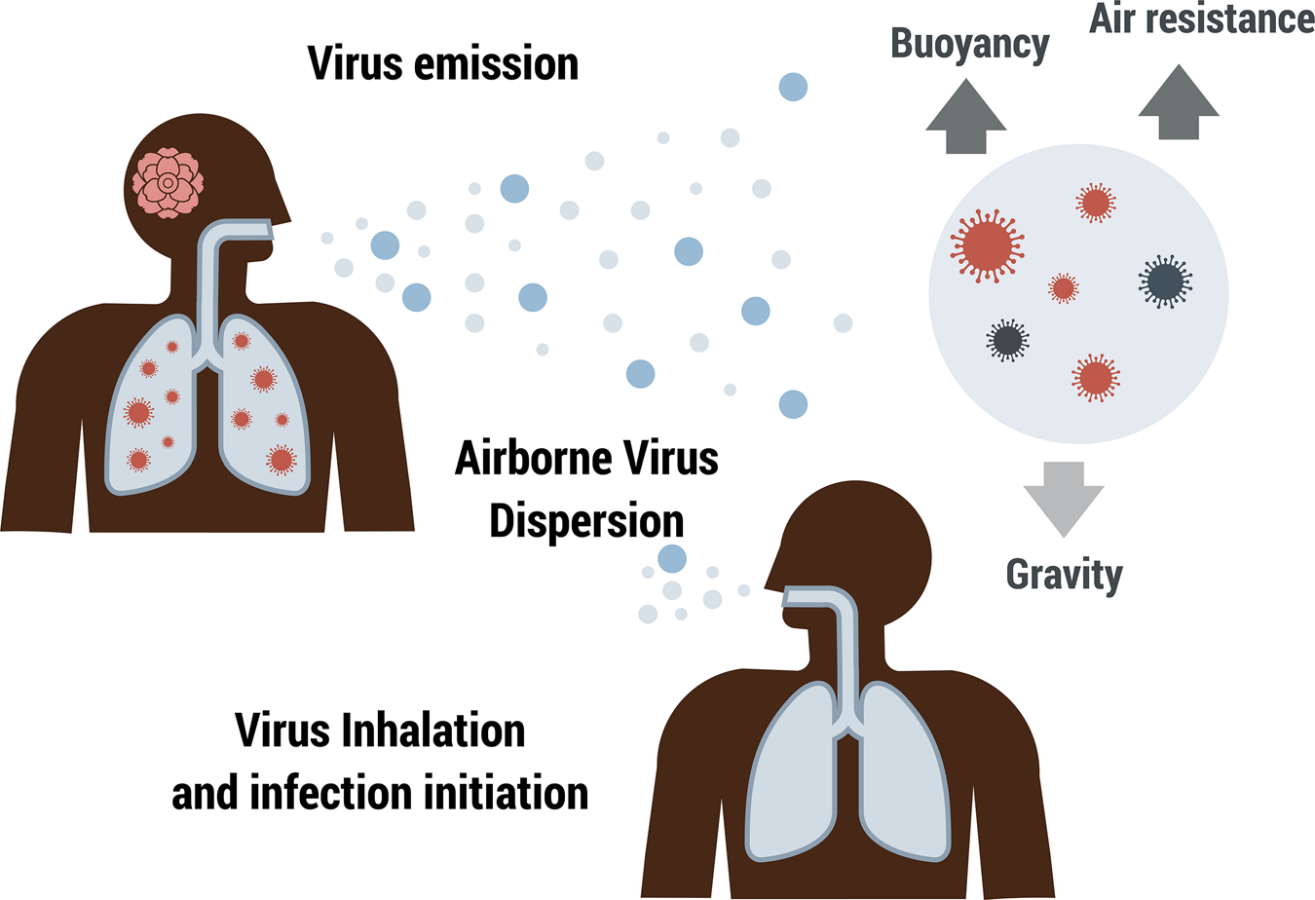
Schematic overview of the airborne transmission model for respiratory viruses in microgravity. The airborne transmission of respiratory viruses is simulated in three main stages. The first stage, *Virus Emission*, involves an infected host releasing virus-laden particles into the air through activities such as coughing, sneezing, talking, or breathing. The second stage, *Airborne Virus Dispersion*, encompasses the spread of these particles through the air. This dispersion is influenced by several factors, including gravitational settling (particles settling out of the air due to gravity), biological decay of the virus (natural degradation of the virus over time), and air management systems (e.g., ventilation and filtration systems that reduce airborne viral concentrations). The third stage, *Virus Inhalation and Infection Initiation*, occurs when a susceptible host inhales the virus-laden particles. The probability of infection is calculated based on the concentration of inhaled particles, their deposition in the respiratory tract, and the host’s immune response. The model takes into account the interplay of these processes to assess the risk of airborne transmission in a given environment.

The risk of airborne transmission of SARS-CoV-2 under microgravity conditions was assessed using the COVID Airborne Risk Assessment (CARA) tool^1^. The CARA tool simulates the spread of respiratory viruses through three main processes (Fig. 4):

#### Virus emission

An infected host releases virus-laden particles into the air through activities such as coughing, sneezing, talking, or breathing. The model quantifies the amount and size distribution of the emitted particles based on the type of expiratory activity and the viral load within the host.

For the baseline scenario, viral load inside the host respiratory tract was sampled from a Gaussian kernel density with a mean of 6.6 and a standard deviation of 1.7 log_10_ copies per mL^39^. Particle size distributions were modeled using the Bronchiolar–Laryngeal–Oral (BLO) model, which describes expiratory aerosols as a multimodal lognormal distribution with three modes corresponding to distinct anatomical origins. Distribution parameters were adapted from Johnson et al.^40^, with an evaporation factor of 0.3 (rather than 0.5 in the original source), yielding modal mean diameters and geometric standard deviations of 0.8 μm (GSD 1.3) for the bronchiolar mode, 1.8 μm (GSD 1.6) for the laryngeal mode, and 3.5 μm (GSD 2.0) for the oral mode. Total particle concentrations were drawn from empirical measurements^41^, and activity-dependent amplification factors were applied for breathing, speaking, or singing/coughing^42^.

#### Airborne virus dispersal

Once emitted, the virus-laden particles disperse through the air. This process is influenced by several factors, including: (1) Gravitational settlement: particles settle out of the air due to gravity, with larger particles settling faster than smaller ones. (2) Biological decay: the natural degradation of the virus over time, which depends on environmental conditions such as temperature and humidity. (3) Air management systems: ventilation and filtration systems that reduce airborne viral concentrations by replacing contaminated air with clean air and capturing virus-laden particles.

#### Virus Inhalation and infection initiation

A susceptible host inhales the dispersed virus-laden particles continuously over the exposure period. The inhaled viral dose depends on the airborne concentration of particles, the host’s breathing rate, the efficiency of particle deposition in different regions of the respiratory tract, and mask efficiency. Breathing rates were sampled from lognormal distributions and varied across physical activities. Particle deposition efficiencies were assumed to vary with size, with smaller particles more likely to reach the alveolar region.

The effective viral dose further accounts for the proportion of virions that remain infectious, represented by the viable-to-RNA ratio uniformly distributed between 0.01 and 0.60, reflecting uncertainty in viral viability. Host-level immunity factors that reduce susceptibility were also incorporated. Infection risk was then estimated by comparing the inhaled dose with the infectious dose required to infect 50% of exposed individuals (ID50), uniformly sampled between 10 and 100 virions based on coronavirus dose-response data. Together, this framework captures variability in breathing patterns, particle deposition, viral viability, mask use, and immune protection.

A summary of the key input parameters used in our model is provided in Table 1. Detailed equations and additional parameters can be found in the published literature^1^.

**Table 1:** Key input parameters and their distribution descriptors used in the CARA (COVID Airborne Risk Assessment) model for simulating SARS-CoV-2 airborne transmission

Parameter	Value/distribution	Source
Viral load in respiratory track (*v*)	Gaussian kernel density with mean and SD of 6.6 and 1.7 log_10_ copies per mL.	^39^
Volume of respiratory particles emitted per exhale volume: *E*_*p*_(*D*)	${E}_{p}\left(D\right)=\,{N}_{p}\left(D\right){V}_{p}\left(D\right)\left(1-{\eta }_{{out}}\right)$	
Number of particles of diameter *D* (*N*_*p*_*(D)*)	${N}_{p}\left(D\right)=\,\frac{1}{D}\mathop{\sum }\limits_{i\in \left\{B,L,O\right\}}\left[\frac{{c}_{i}{f}_{{amp},i}}{\sqrt{2\pi }\,{\sigma }_{i}}\exp \left(-\frac{{\left(\mathrm{ln}D-\,{\mu }_{i}\right)}^{2}}{2{\left({\sigma }_{i}\right)}^{2}}\right)\right]$	
The mean (${\mu }_{i}$) and standard deviation (${\sigma }_{i}$) of the natural logarithm of the diameter		^1,40^
B-mode	0.99 (SD 0.26)	
L-mode	1.39 (SD 0.51)	
O-mode	4.96 (SD 0.59)	
Total particle emission concentrations (*c*_*i*_)		^41^
B-mode	0.06 (cm^−3^)	
L-mode	0.2 (cm^−3^)	
O-mode	0.001 (cm^−3^)	
Amplification factor (*f*_*amp,i*_), *i* ∈(*B*, *L*, *O)*		^42^
Breathing	(1, 0, 0)	
Speaking	(1, 1, 1)	
Singing and shouting	(1, 5, 5)	
Breathing rate (*B*)		EPA Exposure Factors Handbook^47^, data from ref. ^48^
Seated	Lognormal(0.51, 0.043^2^)	
Standing	Lognormal(0.57, 0.043^2^)	
Light activity	Lognormal(1.25, 0.12^2^)	
Moderate activity	Lognormal(1.78, 0.34^2^)	
Heavy activity	Lognormal(3.30, 0.72^2^)	
Infectious dose (ID50)	Uniform [10, 100] virions	
Viable-to-RNA virus ratio	Uniform [0.01, 0.60]	

Parameters include viral load distributions, particle emission characteristics from the Bronchiolar-Laryngeal-Oral (BLO) model, breathing rates for different physical activities, and infection dose-response values. Distribution types and statistical descriptors (mean, standard deviation, or range) are provided for stochastic variables used in Monte Carlo simulations.

### Simulation of microgravity environment

In this section, we describe the environmental settings used to simulate a microgravity environment, such as that found on the International Space Station (ISS). The ISS orbits Earth in a microgravity state, where gravity is not zero but ranges from 10^−3^ to 10^−6^ g^43,44^. This microgravity environment significantly influences the behavior of respiratory particles, as their motion is determined by the interplay of several forces, including buoyancy, air resistance, and the reduced gravitational force. By balancing these forces, the duration that respiratory particles remain suspended in the air (*t*) under microgravity conditions can be determined using the following formula:

1 $$t=\frac{h}{\frac{\left({\rho }_{p}-{\rho }_{\mathrm{air}}\right){\left({d}_{\mathrm{evap}}{\times 10}^{-6}\right)}^{2}g}{18{\mu }_{\mathrm{air}}}},$$

where *g* is the effective gravitational acceleration experienced in the microgravity environment, *h* represents the height from the ground to the mouth of the infected individual, ${\rho }_{p}\,\mathrm{and}\,{\rho }_{\mathrm{air}}$ are the mass densities of the respiratory particle and air, respectively, ${d}_{\mathrm{evap}}$ is the diameter of the particle after evaporation, expressed in micrometers, and ${\mu }_{\mathrm{air}}$ is the dynamic viscosity of air.

Variations in gravity significantly affect the time respiratory particles remain suspended in the air, which in turn influences the concentration of viruses in the air. Our study investigates gravity within the range of 10^−3^ to 10^−6^ g. However, for the specific values of 10^−4^ g, 10^−5^ g, 10^−6^ g, and 0, the results showed minimal differences (see Supplementary Fig. 1). Therefore, we chose to use the 10^−6^ g value for our analysis, as it represents the most conservative estimate of the microgravity environment on the ISS.

Due to the prolonged suspension duration in microgravity, respiratory particles can remain airborne even if they are as large as 100 µm. Consequently, we consider respiratory particles across the entire range of inhalable sizes (1–100 µm^45^) in this context, as opposed to focusing only on smaller particles that remain airborne for extended periods under Earth’s gravity.

The habitable sections of the ISS consist primarily of a series of interconnected cylindrical modules with varying volumes^24^. These include the U.S. Laboratory (Destiny, ~123 m^3^), European Research Laboratory (Columbus, ~109 m^3^), Russian Service Module (Zvezda, ~180 m^3^), Node 1 (Unity, ~80 m^3^), Node 2 (Harmony, ~97.3 m^3^), and Node 3 (Tranquility, ~97 m^3^). The size of these modules directly affects the concentration of viruses within them. For this study, we simulated conditions based on the volume of the Columbus module, as it represents a mid-sized module on the ISS.

Other environmental parameters aboard the ISS are carefully controlled to ensure the health and safety of astronauts during their missions. The temperature is maintained between 20 °C and 25 °C, while humidity levels are controlled within a range of 30% to 60% to optimize crew performance and ensure thermal comfort^46^. Although cosmic radiation levels are higher in space compared to Earth’s surface due to the absence of atmospheric protection, the ISS is equipped with shielding to protect astronauts from harmful radiation^36^. Additionally, advanced air filtration and circulation systems are employed to ensure clean air for the crew, essential for maintaining a healthy breathing environment^11^.

## Supplementary information

Supplementary information

## Data Availability

All data generated or analyzed during this study are included in this published article and its supplementary information file.
