# Supplementary material for: Modeling the risk of airborne transmission of respiratory viruses in microgravity
Source: NPJ Microgravity. 2026 Apr 1;12:44. doi: 10.1038/s41526-026-00590-4 (PMC13201566; doi:10.1038/s41526-026-00590-4)
Supplement: Supplementary file 1 — Supplementary information [file 41526_2026_590_MOESM1_ESM.pdf]

Supplementary Material

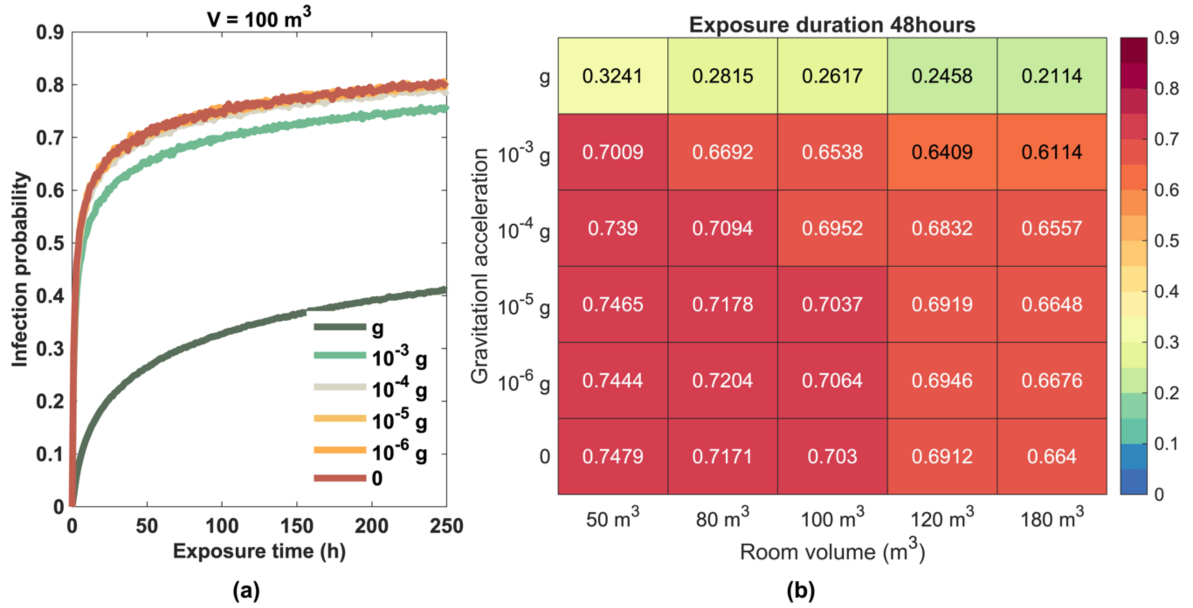

Supplementary Figure 1: Infection probability under microgravity conditions. (a) Infection probability across various gravity levels as a function of exposure time. **(b)** Infection probability across different gravity levels and room volumes after 48 hours of exposure.
